# Efficacy of tailored-print interventions to promote physical activity: a systematic review of randomised trials

**DOI:** 10.1186/1479-5868-8-113

**Published:** 2011-10-17

**Authors:** Camille E Short, Erica L James, Ronald C Plotnikoff, Afaf Girgis

**Affiliations:** 1 School of Medicine and Public Health, Priority Research Centre for Health Behaviour, Priority Research Centre for Physical Activity and Nutrition, University of Newcastle, Callaghan, Australia; 2 School of Medicine and Public Health, Priority Research Centre for Physical Activity and Nutrition, Priority Research Centre for Health Behaviour, University of Newcastle, Callaghan, Australia; 3 School of Education, Priority Research Centre for Physical Activity and Nutrition, University of Newcastle, Callaghan, Australia; 4 Ingham Institute for Applied Medical Research, South Western Sydney Clinical School, University of New South Wales, Liverpool, NSW, Australia

## Abstract

**Objective:**

Computer-tailored physical activity interventions are becoming increasingly popular. Recent reviews have comprehensively synthesised published research on computer-tailored interventions delivered via interactive technology (e.g. web-based programs) but there is a paucity of synthesis for interventions delivered via traditional print-based media in the physical activity domain (i.e. tailored-print interventions). The current study provides a systematic review of the tailored-print literature, to identify key factors relating to efficacy in tailored-print physical activity interventions.

**Method:**

Computer-tailored print intervention studies published up until May 2010 were identified through a search of three databases: Medline, CINAHL, and Psycinfo; and by searching reference lists of relevant publications, hand searching journals and by reviewing publications lists of 11 key authors who have published in this field.

**Results:**

The search identified 12 interventions with evaluations reported in 26 publications. Seven out of the 12 identified studies reported positive intervention effects on physical activity behaviour, ranging from one month to 24 months post-baseline and 3 months to 18 months post-intervention. The majority of studies reporting positive intervention effects were theory-based interventions with multiple intervention contacts.

**Conclusion:**

There is preliminary evidence that tailored-print interventions are a promising approach to promoting physical activity in adult populations. Future research is needed to further identify key factors relating to efficacy and to determine if this approach is cost-effective and sustainable in the long-term.

## Background

Participation in physical activity (PA) is well recognised as an important and modifiable determinant of both psychosocial and physiological health. To date, research on PA emphasises the health benefits associated with participating in regular moderate-vigorous aerobic activity and strength training over one's lifetime [1-3]. There is also recent evidence to indicate that prolonged sedentary behaviour, such as sitting, may be an independent determinant of health, with prolonged sitting associated with ill health regardless of total leisure time activity [4-6].

Despite the known benefits of maintaining an active lifestyle, many people living in industrialised societies are considered to be insufficiently active to induce health benefits [7,8]. In 2000, physical inactivity was estimated to account for 1.9 million deaths world-wide and 19 million disability-adjusted life years [9]. As such, it is not surprising that physical inactivity has been labelled as one of the biggest public health problems in the 21^st ^century [10]. A key challenge is to develop appealing and effective PA programs that can be provided in a cost-effective and sustainable manner. Several reviews have suggested that computer-tailored interventions, that utilise technology to provide individuals with customised health behaviour advice and feedback, offer a promising approach to physical activity promotion [11-20]. These interventions are distinct from (yet commonly confused with) generic and targeted interventions because they are aimed at individuals (within a defined population) rather than a population group (generic) or subgroup (targeted) [11]. Since the last decade, the medium for computer-tailored interventions has become increasingly interactive. Due to advances in technology, there has been a move away from delivering tailored interventions via traditional print media (known as first generation interventions) towards delivering interventions via interactive technology, such as websites or mobile devices (known as second and third generation interventions, respectively [15,17]).

Second and third generation interventions have been put forth as more promising approaches due to the enhanced potential to provide real-time and interactive feedback to an infinite number of participants [13,21]. However, whether these benefits translate into enhanced efficacy is unclear. A recent systematic review [15] examining the efficacy of these latter generation interventions reported that 14 out of 17 included interventions were efficacious in changing PA behaviour, but only 7 of these were more efficacious than the control condition (all of which were wait-list control or minimal contact interventions). Where interventions were tested against other treatment options (such as non-tailored print materials and non-tailored internet sites), there were no significant between group differences. There have also been concerns about the external validity of these latter generation interventions, with studies reporting frequent problems recruiting, sustaining engagement and retaining participants [15]. As a result, more intensive web-based interventions have been recommended, such as utilising prompts through other mediums and ensuring websites are continuously updated and contain dynamic and interactive material [15]. Whilst these interventions undoubtedly do hold great public health promise it seems premature to outcast first-generation print-based interventions at this point.

First, there is no evidence that latter generation interventions are more efficacious than traditional print-based approaches. To date, only one study [22] has compared the relative efficacy of a first and second generation intervention in the PA domain and no significant differences in physical activity outcomes were found. Likewise, a recent meta-analysis [19] found no significant differences of the efficacy of computer-tailored interventions based on delivery channel and concluded that both print and web-based channels can be effective means of health communication.

Second, there are benefits and strengths of the tailored-print approach that should be considered: (1) Tailored-print approaches are likely to have a wider reach and acceptability in populations that are known to have low access and use of the internet, such as people living in rural or remote areas, individuals with lower socio-economic status and older adults [23]. Of note, tailored-print strategies may play a special role in secondary/tertiary prevention, where the above characteristics (e.g. older age) exist in a large proportion of the target group (e.g., majority of cancer survivors are over 65 years of age and cite a preference for print-based interventions [24]) and where there are existing support structures in place that can provide the necessary man power to implement interventions (e.g. The Cancer Council);(2) In times where personal letters are scarce and emails are rife, people may perceive the real novelty lies in receiving a tailored letter. According to the Elaboration Likelihood Model [25], which is often given as the rationale for why tailoring works [11], this perception of novelty could lead to more elaborate processing of the tailored material. There is some evidence that this may be the case, with one study reporting participants had a greater recall of mailed print materials compared to an interactive website [26]. This may also explain why retention for tailored web-based programs is generally poor [15], with the novelty of tailored-websites potentially low compared to other competing sites such as Facebook; (3) If intervention developers are to consider individual preference for delivery mode, there are individuals who report preferring print-based interventions [27,28]. As there is good evidence that tailoring print materials enhances efficacy [11,18], it seems justified that intervention developers may provide tailored-print materials to individuals preferring print delivery modes. However, the same is not true for web-based interventions, with minimal evidence that tailoring websites further enhances efficacy in comparison to non-tailored websites [15,29],

Third, interventions may be more efficacious in changing PA behaviour if first and latter generation interventions are combined to form mixed modal interventions. There is evidence that distance-based interventions are more likely to be effective if more than one delivery mode is used [30] and it has already been suggested that including prompts through other mediums may help improve retention rates for tailored-web-based interventions [15].

Hence, the relative 'promise' of the different approaches stems beyond the time taken to deliver feedback and is likely to be dependent on a number of factors, including the aim of the intervention and the population targeted. In light of this, intervention developers should base their decision on which delivery method or combination of delivery methods are most appropriate by using an intervention development framework, such as intervention mapping [31].

Whilst the evidence for second and third generation approaches in the PA domain has been recently reviewed in a well-conducted systematic review [15], the evidence on tailored-print approaches in the PA domain needs updating. The last comprehensive review was conducted considerable time ago [13] and did not focus on tailored-print physical activity interventions specifically. Likewise, meta-analyses have been conducted but have included other health behaviours [16] and/or other tailoring approaches in the analysis [19]. Reviews that have focused specifically on tailored-print physical activity interventions have been narrative in nature and were conducted over a decade ago [18,32,33]. Whilst these reviews provide some insight into how efficacious tailored-print interventions are and some of the key strategies related to efficacy, none provide a comprehensive overview of the state of the evidence in the PA domain and none provide sufficient information to serve as a guide to those wishing to develop tailored-print interventions.

The primary purpose of this review is to evaluate the evidence for tailored-print interventions in changing PA behaviour, inclusive of aerobic, strength and prolonged sedentary behaviour. Given the known heterogeneity of tailored interventions, this systematic review (1) describes the available evidence and (2) the key factors relating to efficacy. This approach is recommended, rather than a meta-analysis, when there is significant heterogeneity of studies [34]. The secondary purpose of this review is to synthesise the literature in a way that will be valuable to intervention developers.

## Method

### Search Strategy and Data Sources

First, studies were identified through a structured electronic database search of all publication years (until May 2010) in Medline, CINAHL, and PsycInfo. The following search strings were used: (Physical activit* or exercise or motor activity or leisure activities or incidental activity or physical inactivity or sedentary behavio*) AND (Tailor* or expert system or print or message) AND (education or behavio*). These strings were further limited to 'adults' (18 years or older) and English language papers. Second, reference lists of relevant publications were scanned for studies not identified in the search process. Third, journals that published a large number of tailored health education articles were identified by sorting via journal name in endnote. All issues of six selected journals (*Preventive Medicine, Annals of Behavioural Medicine, Health Education Research, International Journal of Behavioural Nutrition and Physical Activity, Patient Education and Counselling *and *Health Psychology) *were searched electronically using Tailo* and physical activit* as key words. Finally, internet searches were conducted using the names of 11 key authors who have published in this domain.

### Study selection criteria

Studies were eligible for inclusion in this review only if they examined at least one computer-tailored print intervention designed to promote PA and/or reduce sedentary behaviour in adults. Interventions were considered 'computer-tailored' if advice was generated for a specific person based on information derived from individual assessment using a computerised system [35]. An intervention was considered to be 'tailored-print' if it involved the delivery of tailored written materials.

Studies were excluded if they: 1) delivered the computer tailored-print intervention in combination with non-print intervention strategies (eg tailored-print plus telephone counselling), hence the efficacy of the tailored-print component alone could not be isolated; b) did not include an appropriate comparison condition; or c) did not measure PA behaviour as a study outcome.

Initially, articles were assessed for eligibility by a single reviewer (CS) based on the study title. After this initial cull, study abstracts were assessed independently in an unblinded standardised manner by 2 reviewers. Findings were compared and disagreements between reviewers were resolved by consensus.

### Data extraction

Previous published reviews [13,15,16,19] were used as a guide for reviewing selected studies and specific intervention characteristics identified as being associated with behaviour change in computer-tailored interventions were extracted. These characteristics included the (1) theory(s) and/or model(s) used to develop the intervention; (2) variables used to tailor messages; (3) format and content of the print materials; (4) frequency and duration of the tailored information being delivered; (5) number of behaviours targeted.

Key methodological characteristics of the identified studies were also extracted, including: the country where the study was conducted, size and source of the study population, eligibility criteria, study design, comparison group, the primary outcome measures and follow-up period. Follow-up periods were divided into three categories: short term (< 3 months), medium term (3-6 months), and long term (> 6 months). The methodological quality of each study was assessed independently by two reviewers using the McMaster quality assessment tool for quantitative studies developed by the Effective Public Health Practice, Canada [36]. Disagreements were resolved by consensus.

## Results

### Study selection

The initial search of the electronic databases yielded 2107 publications, which were reduced to 219 following review of the titles by one reviewer (CS). After removing duplicates and reviewing the abstract (by two independent reviewers), 25 articles met the inclusion criteria for this review and reference checking identified one additional paper. The electronic search of specific journals and search of selected authors did not yield any new papers.

**Figure 1:**
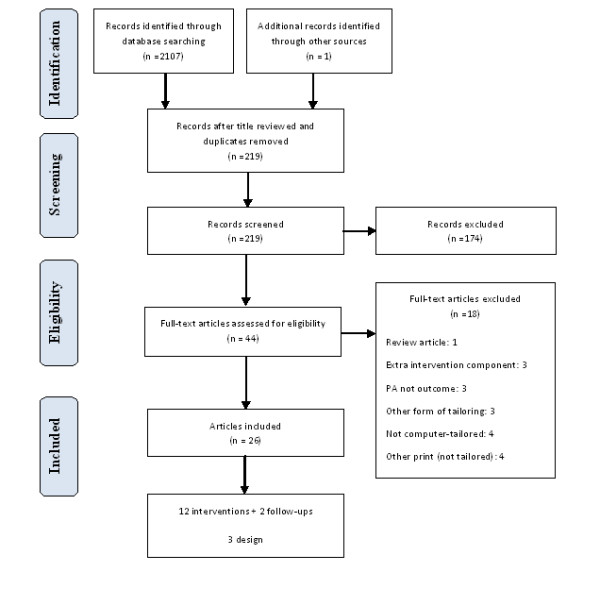
**PRISMA flow diagram summarising selection process**.

A total of 12 interventions [21,22,37-46] were reported in 26 publications [21,22,37-62]; with two [59,62] describing the long-term follow-up of interventions [40,46]; nine describing sub-analyses, including mediation analyses [50,51,54,58,61], moderator analyses [57] and cost effectiveness [52,55]; and three [47-49] describing the study design in additional detail (Figure 1).

**Figure 2:**
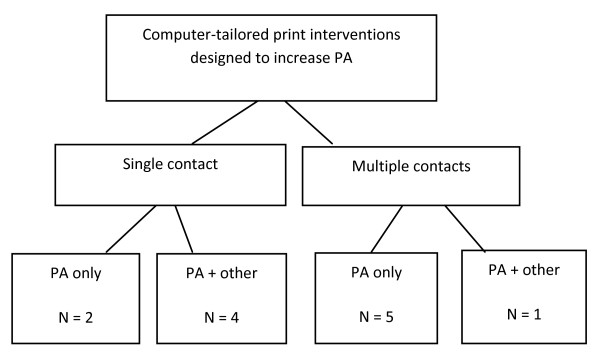
**Categorisation of studies sourced**.

The studies sourced were categorised by: 1) whether the tailored feedback was delivered in a single-contact (referred to as non-iterative) or via multiple contacts (referred to as iterative); and, 2) whether the studies focused on a single behaviour (PA only) or multiple behaviours (PA plus other; Figure 2).

Table 1 (additional file 1) provides a detailed summary of the characteristics of all of the reviewed studies.

### Study Characteristics

Six of the identified studies tested single contact interventions and six tested multiple contact interventions (Figure 2). Of the multiple contact interventions, four [22,40-42] were related, testing an adapted version of the intervention (developed by Marcus et al 1998 [40]) and/or its trial in different settings. The majority of the multiple-contact interventions focused on the promotion of PA alone, whilst most of the single-contact interventions focused on the promotion of multiple health behaviours, including PA (Figure 2). The type of PA targeted ranged from aerobic exercise [39] to activities of daily living, including those performed at a light intensity [22,37,38,40,41,43,44,46]. The majority of studies focused on promoting participation in moderate-vigorous PA. No studies promoted strength training or reductions in unbroken sedentary behaviour (see Table 1, additional file 1).

The majority of the studies were conducted in North America [21,22,37,39-42] and the Netherlands [38,43-45] with one study conducted in Belgium [46]. Participants were recruited via advertisements, primary health care and health education organisations. The majority of studies recruited "at risk" individuals, including adults who were sedentary [22,37,40-43], overweight [21], patients [39] or older [45], with only three studies recruiting from the general population [38,44,46]. Study samples ranged from 194 to 2827 participants with the majority of participants being female, middle-aged and having completed at least a high school education. In studies that reported ethnicity [21,22,37,39-42], the majority of participants were reported as white.

### Intervention Characteristics

#### Comparison group

Six studies [21,37,38,40,42,44] compared tailored print materials to other non-tailored print materials on the same topic (ie generic materials [21,37,38,40,44] or targeted materials [42]). Five studies [22,39,41,45,46] tested the relative effectiveness of different tailored interventions against a control group. Of these, three tested variations in tailored print interventions [39,45,46] and two compared tailored print interventions to tailored interventions delivered via another method (telephone [41] or internet [22]). Finally, one study [42] compared a single tailored-print group to a control group. Some studies matched the study conditions to varying degrees by controlling for formatting, theoretical underpinnings and the number of contacts (see Table 1, additional file 1).

#### Theoretical Models, Tailoring variables and feedback type

Most of the interventions were informed by The Transtheoretical Model (TTM; [63]) in conjunction with at least one other behaviour change theory (see Table 1, additional file 1). In four studies [38,43-45], an integrated model (I-change model [64]) was used. In other cases, the use (joining) of multiple theories to inform the intervention was based on empirical evidence and expert opinion regarding the determinants of behaviour change. One study [37] relied upon a single theory (TTM) and another [21] made reference to several theory-relevant constructs, without referring to a specific theory.

All studies tailored materials based on psychosocial variables (e.g. perceived barriers), with some also tailoring on behavioural [21,22,38-46], demographic [21] and environmental variables [45]. The feedback type differed between single and multiple contact studies, with multiple contact studies able to provide progress feedback on psychosocial and behavioural variables (not possible in single-contact studies) as well as comparative and evaluative feedback (possible in single-contact studies) about how individuals' health behaviours (e.g. PA, nutrition) compare to national recommendations and to the profiles of other successful individuals.

The majority of studies gave some detail about the content of the tailored materials, such as examples of the actual messages [40,42,43] or a description of the variables that were used to create each message [21,37,38,43-46]. However, most studies did not adequately describe the operationalisation of the tailoring variables (see Table 1, additional file 1). For example, only one study [45], which used an intervention mapping protocol [65], explicitly outlined the theoretical methods and practical strategies that were linked to the tailoring variables used to create each message.

#### Delivery and format of print materials

The majority of tailored print materials were delivered through the mail in either a standard letter or newsletter format [22,37-45]. Delay in delivery of mailed materials, relative to baseline measurement, ranged from 3 days [37] to 4 weeks [39] in the 8 studies reporting this variable. Two studies [21,46] delivered print materials onsite. In one of these studies [21], the materials were generated beforehand based on a telephone interview, but the gap between the interview and the onsite visit was not reported. In the second study [46], participants completed the baseline questionnaire on a computer kiosk onsite, and received the tailored feedback instantly on the screen and were given a print out of the information to take home.

### Measurement of Tailoring Variables

The majority of studies reported some information regarding how many items were used to assess the tailoring variables and the number of response options per item (Table 1, additional file 1). Only three studies [22,40,41] provided psychometrics (ie reliability/validity information) for each item or set of items associated with the tailoring variables; and four [37,38,42,43] provided some psychometric information about their measures for at least one but not all of the variables. Variables relating to the TTM were well-described across studies; those relating to other theoretical frameworks were inconsistently reported.

### Measurement and Primary Outcome Variables

#### Physical Activity

All studies assessed PA behaviour using subjective self-report measures. One study [41] used an objective measure to confirm the validity of the questionnaire (weak correlation) and two [22,41] used an objective measure as a secondary outcome (fitness measured by a graded submaximal exercise treadmill test). Of the self-report measures that were used, nine studies [22,38,40-46] reported that the measure was valid and reliable and three studies [21,37,39] used single-item questions with unknown reliability and validity.

Nine studies [21,22,37,38,40-42,45,46] used continuous primary outcome variables (ie minutes/week [22,38,40-42,46] ; number of sessions per week/month [21,37,45]). Four of these studies [38,40,41,45] also calculated a dichotomous categorical primary outcome variable of whether or not participants were meeting a national health recommendation for PA. Three studies [39,43,44] used a categorical primary outcome variable only (yes/no meeting PA guidelines [43,44]; yes/no exercising > three times a week [39]).

Most studies based outcome assessment on multiple domains of PA (eg leisure, transport, occupation) performed at a moderate intensity or higher, except for one study [39] that only measured aerobic activity and one [46] that included light physical activities as a part of a total PA score (Table 1, additional file 1). Two studies did not specify the intensity of the PA measured [37,39] but specific categories of PA were provided.

#### Follow-up periods

Post-baseline and post-intervention follow-up measures are described in Table 1 (additional file 1). Follow-up periods for single-contact interventions ranged from short-term (1 month) to mid-term (6 months). Multiple contact studies had longer post-baseline follow-up periods ranging from mid-term (3 months) to long-term (12 months) but some of these studies did not include post-intervention measures [22,41]. Post-intervention measures in the multiple contact studies ranged from 3 months [38] to 6 months [59].

### Review of Methodological Quality

Based on assessments by two reviewers using a standardised tool [36], only one [44] of the studies was rated as 'strong', eight [22,37,39-42,45,46] received a global rating of 'moderate' and three [21,38,43] received a global rating of 'weak'. Inter-rater-reliability between the two reviewers was high and all discrepancies were resolved via consensus. Inadequate reporting of randomisation method, consent rates, assessor and participant blinding to study outcomes, and withdrawal differences between study groups were common methodological limitations across studies. All studies relied solely on subjective self-report measures of PA behaviour for the primary outcome. Marcus et al (2007a; [41]) used an objective measure (accelerometer) to confirm the validity of the self-report measure but the correlation coefficient was weak (.32). Marcus et al [48] also reported using an accelerometer to verify responses, but these data were not reported [22]. In three studies [21,37,39] the measures had not been validated and were not as comprehensive (single-item) as the measures used in the other studies (multiple items). Selection bias was a potential issue in nine studies [21,22,38,40-43,45,46] due to a low consent rate and/or the recruitment method (self-referral). Intervention integrity was compromised in the majority of studies [21,37,39,40,44-46,59] by failure to undertake (or report undertaking) intention to treat analyses. Of these studies, dropout rates ranged from 14% [39] to 39% [59] and one study did not report on participant withdrawal [21]. Only five studies [38,39,43-45] reported the magnitude of intervention effects (ie effect sizes). Table 1 (additional file 1) describes the methodological subcomponents that obtained a weak rating for each of the included studies.

### Intervention Effects on Physical Activity

As no studies targeted reductions in unbroken sedentary time or participation in strength training, the following results relate to aerobic PA performed at a light-to-vigorous intensity.

Seven [38,40-42,44-46] studies reported significant short- to long-term positive intervention effects on PA, ranging from 1-24 months post-baseline and 3-18 months post-intervention. In one study [44], the positive effect was defined as a reduction in the decline of PA over the study period (3 months) compared to the control. Where calculated, intervention effect sizes were reported as small (Cohen's *d *ranging from 0.12-0.35; Odds ratio's ranging from 0.82-1.34; [38,39,43-45]) but fewer than half of the studies made this calculation. Five of the studies (out of the seven with positive results) included multiple post-baseline follow-ups [38,40-42,46]. Sustained intervention effects were found in all but one study [42]. In another study [40], sustained effects (at 12 months) were found for meeting PA guidelines but not for minutes/week of PA.

Of the five studies [21,22,37,39,43] that did not find significant positive intervention effects on PA: two [22,37] reported significant increases in PA in all study groups but no significant differences between groups at mid- and long-term; one study [38] found a positive intervention trend that was not significant at mid-term; one study [43] reported significant positive intervention effects at mid-term for motivated participants only; and one study [37] revealed significant increases in participants' preferred type of PA at mid-term but no overall intervention effect on total PA. Only one study [21]reported a negative intervention effect (in a sub-analysis), where participants receiving generic materials that matched their individual characteristics (by chance) increased their PA more than participants receiving (deliberately) tailored print materials at short-term.

### Evaluation of Key Intervention Factors Impacting on Effectiveness

#### Number of contacts

Multiple-contact studies appeared to be more effective in changing PA behaviour than single-contact studies. Only two [43,46] of the six single-contact studies reported the tailored-print interventions as superior to the control group. In contrast, five [38,40-42,45] out of the six multiple-contact studies reported superior intervention effects for the tailored-print condition. The remaining study [22] reported significant intervention effects, but did not find between-group differences between the tailored-print arm and two theory-based internet arms (one tailored and one non-tailored).

#### Number of behaviours targeted

Out of seven studies reporting positive intervention effects, four focused on PA behaviour only [40-42,45] and three targeted multiple health behaviours. This is potentially confounded by the greater number of multiple-contact studies focusing specifically on PA behaviour and the greater number of single-contact studies targeting multiple behaviours (Table 1, additional file 1).

#### Comparison groups

Comparison groups may have partially explained intervention effects. While there were no clear differences between minimal (e.g. generic materials) or no intervention control groups, of exception were the studies testing tailored-print materials against more rigorous interventions (targeted-print materials [42], tailored-telephone calls [41] or a tailored website [22]). Only one of these studies found a significant intervention effect in favour of the tailored-print materials [41]. It is worth noting that in this study, both interventions (tailored print and tailored-telephone calls) produced positive effects at mid-term but only the tailored-print condition produced sustainable effects at long-term. In the other studies comparing tailored print to more rigorous interventions, a marginally significant positive effect was found (compared to the targeted materials) at mid-term but not at long-term [42] and significant increases in PA were found across conditions (tailored-print and tailored-internet and standard internet) but no significant between group diffrerence at mid or long-term were reported [22].

Of the three studies comparing the relative effectiveness of variations in tailored print interventions (varying on one factor) to a control group, significant intervention effects were attributed to differences between the intervention arms and the control group only. That is, intervention effectiveness was not enhanced nor reduced by the inclusion of environmental information [45], action plans [38] or by whether or not information on different behaviours was delivered simultaneously or sequentially [46]. Of note, a significant positive effect of including environmental information in the tailored-print materials [45] was reported in a subsequent paper due to differences in primary outcome variables (ie total weekly days of PA verses total weekly minutes of PA; [57]).

#### Theoretical underpinning

Interventions seemed to be most effective when underpinned at least in part, by either: Social Cognitive Theory, The Theory of Planned Behaviour or the I-Change Model. The use of the TTM alone [37] or the use of no theory [21] may be related to lower efficacy.

#### Delivery delay of print materials

Delivery time may have had an effect on intervention efficacy but it is difficult to draw a clear conclusion due to the lack of available information. Of the seven studies that reported positive intervention effects on primary outcomes, four did not report delivery timeframes of print materials (see table 1). Where delivery time-frames were reported, positive intervention effects were found for studies delivering feedback ranging from immediately up until 2 weeks post baseline.

#### Primary Outcome Variables

There were no clear differences in overall efficacy based on the use of continuous verses categorical dichotomous primary outcome variables. There was some indication that both types of outcome variables may be sensitive to detecting behaviour change at different time-points [40] but this was not the case in the majority of studies that included both types of outcomes [38,41,45].

#### Methodological quality

There were no marked differences in the overall methodological quality between studies reporting significant versus non-significant results. However, studies reporting a positive result were more likely to have used a valid and reliable PA performance measure (Table 1, additional file 1). Overall, the majority of studies reporting positive intervention effects were rated as 'moderate' in methodological quality [40,41,45,46], with one rated as 'strong' [44] and only one rated as 'weak' [38].

### Mediators and Moderators of Intervention Effects

Six studies [21,41,43-46] tested for interaction effects in order to identify possible modifiers. Whilst several modifiers were identified, the direction of modification was inconsistent across studies. For example, where BMI was assessed, one study [46] reported an association between higher BMI and increased PA, two studies [21,45] reported an association between lower BMI and increased PA and one study [44] reported no association. Of importance, there was some indication that intervention effects were not moderated by PA levels at baseline.

Only four studies [21,40,41,45] conducted mediation analyses. Analyses were restricted to variables relating to the TTM and perceptions about the tailored materials. The results of these analyses were inconclusive and provide only minimal evidence that PA increases are mediated by changes in constructs from the TTM (ie self-efficacy, cognitive and behavioural processes, decisional balance).

### Cost-Effectiveness

Only two studies [52,55] reported cost-effectiveness data. These studies were related, testing the same 12 month tailored-print intervention against different conditions (tailored-telephone [55]; tailored-internet [52]). The cost of delivering the tailored-print intervention ($35.81 per month per participant [52]) was consistent between studies. In the study comparing tailored print to tailored telephone calls [55], print was found to be more cost-effective at 12 months in terms of the cost of moving one person out of sedentary behaviour ($955 for the print group and $3, 967 for the telephone group)[55]. Likewise, in the tailored-print versus tailored internet study [52], print was reported as more cost-efficient at 12 months in terms of intervention delivery costs ($439 per participants per year compared to $1470.29). However, it was noted that the internet intervention may be less costly per participant if the number of participants was increased (i.e. assuming the same additional costs for each added participant the internet intervention would be less costly than the print condition when N > 352). Of note for intervention developers, the tailored print and tailored-internet interventions cost $10, 742 and $109, 564 (USD) respectively, to develop.

## Discussion

This systematic review advances the field of knowledge on the efficacy of first generation tailored-print interventions in promoting PA behaviour in adults. Whilst the small number of relevant published studies needs to be considered when drawing conclusions from the review, it provides evidence for the efficacy of tailored-print interventions for enhancing aerobic PA participation in adults. Both single-contact and multiple-contact studies of reasonable methodological quality have demonstrated they can be efficacious in promoting PA behaviour in the mid and long-term. Nevertheless, the magnitude of the effect is unclear and evidence is restricted only to aerobic PA and assessed mostly in the mid-term.

### What do these studies tell us about optimum intervention intensity?

The delivery of more than one tailored-print material seems to be a key determinant of intervention efficacy, with multiple-contact studies showing superior effects compared to single-contact studies. This indicates that more intensive interventions, in terms of both contact and ability to provide relevant feedback, may be more efficacious. Exactly how many tailored-print materials should be delivered and in what timeframe, is difficult to determine due to the heterogeneity of studies, the limited number of effect-size calculations and the lack of post-intervention follow-ups in multiple-contact studies. This finding is consistent with previous research [16,19].

One important consideration, from a public health perspective, is that optimal intervention intensity may be dependent on participant characteristics, with single-contact interventions sufficient for individuals ready or able to make behaviour changes but not for individuals with higher needs. This would explain why positive intervention effects in single-contact studies were limited to those conducted with self-referred healthy adults and not those conducted with sedentary and 'at risk' individuals. Furthermore, this would explain why motivated 'at risk' participants responded more positively to the intervention [43] and why they were more likely to increase PA that they enjoyed [37]. Hence, the search for an 'optimal intensity' may be population and behaviour specific.

### What do these studies tell us about the constructs used to tailor messages?

To date, much remains unknown about what specific aspects of tailoring contribute to the effectiveness of tailored messages. This is known as "the black box of tailoring". In the reviewed studies, tailored messages were primarily composed of messages pertaining to PA behaviour and psychosocial constructs, drawn from a handful of behaviour change theories. Overall, the constructs used to tailor messages between studies were similar but there was some variability in how the constructs were used that may explain the differential intervention effects. For example, the theoretical construct 'stage of change' was used to decide: who would receive information about PA at all [37,39]; which information was emphasised [43]; and how feedback on other constructs, such as self-efficacy, would be delivered [44,46]. The relative effectiveness of these approaches is unclear, although it seems that using the stage of change construct to determine what to emphasise or how to present information is more effective than using it to screen participants. There was also variability in the type of feedback or information given for each construct. For example, behavioural feedback seemed to be more effective when it was based on individual progress rather than when it was based on comparisons with perceived level of activity or current guidelines.

Given that the majority of studies were 'theory-based', they should provide some insight into how tailoring 'works', that is, theory should provide a common description of what is known within an organising system [66]. Unfortunately, in many studies, theory was used as a 'loose framework', with theoretical constructs rather than theory used to guide the development and delivery of the intervention and such constructs not always considered in the analysis and interpretation of study outcomes.

Another factor contributing to the 'black box' of tailoring is the lack of analysis regarding the mediators and moderators of intervention effects. Whilst some studies reported these analyses, there were too few to draw specific conclusions about why tailoring 'worked'. Self-efficacy appears to be an important construct, but the evidence is inconclusive. There was also evidence that tailored-print interventions based on these constructs work equivalently for people with different levels of PA at baseline. This highlights the potential for tailored-print interventions to play a significant role in PA maintenance as well as initiation.

### Generalisability of the findings

Although this is the most comprehensive review of the efficacy of tailored-print interventions to promote PA behaviour change in adults, several factors may impact on the generalisability of its findings. First, the findings are based on a small number of studies (12) of predominantly middle-aged, inactive females. Second, the review did not include grey literature (i.e., unpublished studies), hence publication bias may be an issue. However, given the number of published studies with null findings or small effect sizes, we believe publication bias is unlikely. Third, the included studies were limited to those focused on primary prevention. Several tertiary interventions were identified, but these were excluded because they included additional intervention components that made it impossible to isolate the effects of the tailored-print components. Fourth, it was beyond the resources of this project to include papers published in languages other than English. Finally, the methodological review conducted as a part of this study revealed several methodological weaknesses that should be considered when interpreting the generalisability of our findings. Despite these factors, this review provides significant insight into the state of the evidence and highlights key directions for future research.

### Future directions

Future consideration should be given to (1) the theoretical underpinnings of tailored-interventions; (2) how we can determine which components of tailored interventions are important; (3) the impact of different intervention intensities; (4) the most appropriate comparison groups in tests of intervention efficacy in terms of both PA outcomes and cost; (5) what population parameters, if any, are predictive of success in tailored-print interventions; and finally (6) the type of PA that should be promoted and how it should be measured.

#### A move towards Social Cognitive Theories

All but one of the interventions in this review explicitly referred to the TTM as forming a part of the theory-base for the intervention. This is not surprising, in that the TTM offers an intuitive way to tailor information. However, since many of these studies were conducted, the use of the TTM in the PA domain has become controversial, with suggestions that there is little evidence that stage-based interventions are effective in the long-term [67]. Furthermore, reviews of tailoring research have shown that interventions that are developed based on social cognitive theories are most effective [16,19]. This was supported in this review with studies underpinned by Social Cognitive Theory or The Theory of Planned Behaviour demonstrating more positive effects. Future research should focus on operationalising these social cognitive theories by mapping the theoretically derived determinants (psycho-social constructs) to behaviour change techniques that can be used in a distance-based and tailored setting (see Michie et al. [68,69]). Intervention developers should also consider selecting behaviour change techniques that have known efficacy in terms of positive increases in PA and associated determinants [70-73]. For example, there is increasing evidence that targeting self-regulation constructs is a promising approach [72,74]. Finally, researchers should detail this process so that there is transparency about how the theoretical underpinnings guided the development of the intervention and to determine the extent to which the interventions were tailored.

#### Mediator analyses

Future studies should seek to identify what tailoring components lead to successful outcomes by conducting appropriate mediation analyses and interpreting results (in light of these analyses) and the theory used to guide the development of the intervention.

#### Optimum intervention intensity

Whilst there is growing evidence that multiple contact studies are more efficacious than single contact studies, there is still only limited information about the optimal number of intervention contacts and the optimum delivery schedule in multiple contact studies. Intervention developers should base intervention intensity decisions on what is known about the population and report effect sizes for both immediate and long-term follow-ups.

#### Distance-based intervention alternatives

Due to the predominant use of no-information or generic information control groups and the limited reporting of effect-sizes, the reviewed studies provide only limited information as to whether tailored-print interventions are comparative in efficacy to other promising interventions, such as targeted-print interventions or second and third generation tailored interventions (eg tailored websites, emails, text messages). The comparison between targeted-print materials and tailored-print materials is particularly important. Targeted materials (those aimed at specific subgroups) are less resource intensive (in terms of both time and cost) and may be equally efficacious in promoting health behaviour change, especially when the target group is somewhat homogenous in terms of demographics, psychosocial characteristics and behavioural patterns. As effective print-based interventions are needed, future research should focus on determining the relative effectiveness and cost-benefit of these two approaches. More research is also needed comparing tailored interventions delivered via different channels or mixed model methods (e.g. complete an online survey and receive a tailored letter via the mail). In all cases, comparison interventions should be rigorously developed and theory-based and the costs associated with development and delivery should be reported where possible.

#### Diverse target populations and moderator analyses

The majority of participants included in the trials summarised in this review were white middle-aged sedentary women. Future research should focus on whether or not tailored-print approaches are effective in other target populations, such as in tertiary prevention, with younger populations or with already active individuals (to facilitate maintenance). Future research should aim to test the generalisability of our results by testing the efficacy of tailored-print interventions in understudied and diverse populations and by conducting moderator analyses that highlight which specific sub-groups interventions were most efficacious in. These analyses could then inform the development of both tailored and targeted intervention materials.

#### Addressing common problems in PA research

Future studies aiming to promote PA participation should consider targeting both aerobic and strength based physical activities. Furthermore, in light of the new evidence surrounding sedentary behaviour [4-6], PA could be promoted in a way that breaks up time spent sitting or laying down during waking hours. This requires a shift in focus from looking at the total amount of PA to the pattern of activity each day and a subsequent change in measurement tools.

All of the studies included in this review relied upon subjective measures of PA. Future studies should include objective and sensitive PA measures; for example accelerometers and pedometers (with a diary) may be particularly useful, especially for determining the pattern of PA behaviour. Future studies should also consider the length of follow-up necessary to inform policy makers and health practitioners on the sustainability of the effects. Several of the reported multiple-contact studies did not include post-intervention follow-ups; and where they were included, they were of no longer than 6 months post-intervention. Given the tendency for relapse once intervention support is withdrawn, the follow-up periods in these studies are inadequate for assessing the long-term efficacy of the intervention. Furthermore, given that PA benefits are obtained from sustained and regular participation, future studies should be powered to assess multiple follow-up periods over an extended period of time, inclusive of short, medium and long-term follow-up periods.

## Conclusion

There is preliminary evidence that tailored-print interventions are a promising approach to promoting PA in adult populations. Future research is needed to determine if this approach is cost-effective and sustainable in the long-term, especially in comparison to other distance-based interventions showing potential, such as targeted-print interventions or other tailored interventions. This systematic review can serve as a guide to researchers and practitioners interested in understanding and/or developing tailored interventions in the PA domain.

## Conflicts of interest

The authors declare that they have no competing interests.

## Authors' contributions

CS participated in the design of the study, reviewed the literature and drafted and revised the manuscript. EJ and RP participated in the design and coordination of the study, and revised and edited the manuscript. AG participated in the co-ordination of the study and revised and edited the manuscript. All authors have read and approved the final manuscript

## Supplementary Material

Additional file 1 **Table 1: Summary table of study and intervention characteristics**. *The data provides a summary of each study regarding the following areas: Context/setting and sample characteristics; Intervention characteristics & control condition; Study design & evaluation Method; Outcome measures; and Key Findings*. Click here for file
