# Supplementary material for: Efficacy of tailored-print interventions to promote physical activity: a systematic review of randomised trials
Source: Int J Behav Nutr Phys Act. 2011 Oct 17;8:113. doi: 10.1186/1479-5868-8-113 (PMC3214130; doi:10.1186/1479-5868-8-113)
Supplement: Additional file 1 — Table 1: Summary table of study and intervention characteristics. The data provides a summary of each study regarding the following areas: Context/setting and sample characteristics; Intervention characteristics & control condition; Study design & evaluation Method; Outcome measures; and Key Findings. [file 1479-5868-8-113-S1.DOCX]

Table 1. Summary of the design and intervention characteristics of the reviewed studies

| **Single-contact studies** | | | | | | |
| --- | --- | --- | --- | --- | --- | --- |
| **Study**  *Study and sub-study references*  *Country* | ***Change of Heart Study***  *Bull et al 1999* [37]  *North America* | Smeets et al 2008 [43]  *The Netherlands* | Kreuter etal 1996 [39]  *North America* | Kreuter et al 2000 [21]  Sub-analysis [61]  *North America* | Smeets et al 2007 [44]  *Netherlands* | **FAITH Project**  Vandelanotte et al 2005 [46]  Follow-up [62]  Feasibility [60]  *Belgium* |
| **Sample** | Patients from family medical clinics in contemplation or preparation stage of change (N = 272) | Sedentary adults (not meeting PA guidelines) recruited via mass mail out with addresses obtained from telephone company (N = 487). | Patients from family medical clinics (N =1317) | Overweight adults interested in losing weight recruited from newspaper advertisements (N=198) | Adults recruited from large companies, door-to-door newspaper articles and letters sent to addresses obtained from telephone company (N =2827) | Adults recruited using local media, posters, leaflets and email (N=771) |
| **Behaviour**  **Targeted** | PA (leisure & PADL) | PA  (transport, sport, leisure) | PA (aerobic); Smoking; seat belt use; fat intake; mammography screening; pap testing; cholesterol testing. | PA (moderate);  Diet (low-fat alternatives) | PA (Leisure, commuting, household, sport);  Nutrition  (F&V, Fat intake);  Smoking | PA (work, transportation  ,household, leisure)  Fat intake |
| **Comparison group (s)** | Personalised print (PA);  General print (PA);  Usual care;  Controlled for formatting | No Info | Risk feedback;  No Info | Generic print (weight loss);  Generic print controlled for formatting (weight loss) | Generic print (PA, Nutrition, Smoking) | No Info  Varied delivery condition of tailored intervention |
| **PA primary outcome measures** | Frequency of PA for 30+ min a day in the past week for 8 different categories of PA. | Self-report SQUASH questionnaire. | Single item | Single items | Self-report SQUASH questionnaire. | Self-reported 31-item IPAQ. |
| **PA primary outcome variables** | Total number of Sessions | Meeting recommendation | Engaging in aerobic exercise 3 times a week  (yes/no) | Frequency of Moderate PA for 30 + mins | Meeting the Dutch recommendation for PA  Action moments  (Mins/wk x intensity). | Total PA(Mins/wk)  Mod-High intensity PA (Mins/wk). |
| **Follow-up (post-baseline)** | 3 months | 3 months | 6 months | 1 month | 3 months | 6 months  24 months |
| **Results** | Increases in PA sessions across all groups but no differences between study groups on total number of PA sessions at mid-term. Significant intervention effect at mid term for preferred PA type. Marginally significant effects for PADL. | No post-test group differences for meeting the recommendation at mid-term  *Significant intervention effects for motivated participants  **ORs reported** | Marginally significant intervention effect at mid term for change in at least one behaviour (OR =1.18, *p* < 0.06).  More post-test increases in aerobic activity in tailored group compared to other groups but not significant (OR =1.34, p = 0.106). **ORs reported** | No significant intervention effects.  **Negative effect** Significant effect for non-tailored “good –fitting” materials for PA in the short term | Significant intervention effect at midterm for PA (intervention reduced the decline of PA).  **ES reported**  Small (d = 0.12). | Significant intervention effects found for both interventions compared to the control group for total PA at mid-term. No significant differences between interventions [1] (simtailored = +173 mins/wk; seqtailored = +213 mins/wk; control =+14 mins/wk) .  No significant effect for mod-high PA at mid-term (simtailored = +61mins/wk; seqtailored = +93mins/wk; control =+45 mins/wk). Intervention effects were maintained at two years follow-up. |
| **Methodological Quality** | **Moderate**  Baseline PA not reported.  Validity of measure not established. | **Weak**  Low consent rate (30%)  Withdrawals reported but high drop out. | **Moderate**  Validity of measure not established  No intention to treat analysis. | **Weak**  Recruited via self referral.  Baseline PA not reported.  Validity of measures not established.  Withdrawal not reported.  Intention to treat analysis not reported. | **Strong**  No intention to treat analysis. | **Moderate**  Participants recruited via self referral  No intention to treat analysis and drop out per intervention group not described. |
| **Theory** | Stages of Change | I-Change Model | Stage of Change  Health Belief Model | Theory not explicitly stated by the author. | I Change | Theory of planned behaviour  Stage of Change |
| **Variables used for tailoring**  Key  Items were described *  Psychometrics given **  No description - | Stage of change **  Exercise goal*  Motives for exercise *  Perceived barriers *  Preferences for PA * | Stage of change (*calculated*) *  Awareness of PA *  Pros and Cons of PA *  Social influence *  Suggestions to cope with barriers (self-efficacy enhancing info)*  Preferred activity –  PA score ** | Stage of change -  Perceived barriers -  Reasons for wanting change -  Perceived health risks -  Perceived health benefits -  Self-efficacy -  Past attempts and failures to change behaviour –  PA score * | Beliefs –Motives – Barriers –Triggers –  Self-efficacy - Goal-setting - Preferred media and learning style-  BMI –  Gender –  PA score-  *Measures described as: close-ended questions with 2-8 response options. | Awareness -  Attitudes *  Self-efficacy*  Stage of change *  Social influence of partner * Barriers –  PA score** | Stage of change -  Intentions –attitudes-  self-efficacy- social support- knowledge- benefits/barriers of behaviour change-  PA score**  *Feasibility trial describes assessments as “based on previous research” but details not given. |
| **Operationalised?** | Stage of change: Only individuals at contemplation and preparation given advice about PA.  Examples given of how tailoring variables used. | Stage of change score determined which parts of feedback received the most attention.  Examples given | Received tailored information when a behaviour was a problem and they were interested in changing it.  Citation given for types of message example but none relate to PA | lists broad content area of weight loss materials  Provides example of how tailoring was based on multiple responses. For example 8 possible barriers. | SOC message differed according to attitude and self-efficacy  Suggestions to increase PA based on barriers and social environment | How each theory was used described. Constructs in TPB not described.  Stage of change considered in two ways: -content differed between stages.  -Way info was presented differed between stages |
| **Delivery method & delay** | Mailed within 3 days | Mailed within a few days to 2 weeks following baseline | Mailed within 2-4 weeks of baseline | Onsite | Mail - time not described | Print out onsite  Immediate feedback |
| **Format of materials** | 2 page letter | 3-5 page Letter | Health Risk Assessment + Single page for every problem behaviour they were interested in changing.  Text and graphs | 4 pages of text  Multi-colour printing | 5-11 pages(s) letter  Cartoons, text and graphs. | Displayed on computer screen, read, printed and taken home (5-6 pages) |
| **Content of Print Materials** | Feedback based on psycho-social constructs  Feedback type not specified | Tailored feedback based on Psycho-social constructs and behaviour scores  Evaluative feedback:  PA score compared to self-assessed PA level and recommended minimum level.  Descriptive feedback: psycho-social constructs | Contents consist of health risk appraisal and tailored information  Feedback based on psychosocial and behavioural variables.  Descriptive feedback: List risk behaviours.  Evaluative feedback: List which behaviours need to be changed and suggest change strategy based on psycho-social construct. | Tailored Feedback based on psychosocial, behavioural and  Demographic variables  Evaluative feedback: behaviour change strategies based on psycho-social variable. Examples not given for how behavioural (PA level) feedback was delivered. | Tailored feedback based on psychosocial and behavioural variables  Evaluative: PA score compared to self-assessed PA level and recommended minimum level. Misconceptions addressed. Behaviour change strategies based on psycho-social variables. | Tailored feedback based on psychosocial and behavioural variables.  Evaluative: PA level compared to recommendation. Tips given to increase PA in different settings based on psycho-social variables. |
| **Delivery Schedule** | Baseline | Baseline | Baseline | Baseline | Baseline | Baseline |
| **Tailoring assessment period** | Baseline | Baseline | Baseline | Baseline | Baseline | Baseline |
| **Interpretation of results based on theory?** | Focus in on variability rather than theory. | ELM  Motivation | HBM provided a useful framework for identifying important intervention components to include in tailored materials.  But no analysis into how they mediated intervention effects | Cognitive responses to intervention material coded based on ELM and past research. | Tested moderators of effectiveness | Sequential vs simultaneous feedback discussed in context of Stages of change.  Suggest possible cognitive process for multiple behaviour change and Socio-demographic variables related to behaviour change explored. |
|  |  |  |  |  |  |  |

| **Multiple-contact studies** | | | | | | |
| --- | --- | --- | --- | --- | --- | --- |
| **Study**  *Study and sub-study references*  *Country* | **Jump Start**  Marcus et al 1998 [40]  Follow-up [59]  Sub-analysis [50]  *North America* | **Women’s wellness project**  *Adapted from jumpstart*  Napolitano et al 2006 [42][2]  *North America* | **Project STRIDE**  *Adapted from Jump Start*  Marcus et al 2007a [41]  Sub-analysis [55] [54] [51]  Design [48]  *North America* | **STEP into Motion**  *Adapted from jump start*  Marcus et al 2007b [22]  Sub-analysis [52]  Design [47]  *North America* | **Active Plus**  Van Stralen et al 2009 [45]  Sub-analysis [57-58]  Design: [49]  *The Netherlands* | De Vries et al 2008 [38][3]  *The Netherlands* |
| **Sample** | Sedentary adults recruited through newspaper advertisements (N = 194) | Sedentary women recruited via info booths in supermarkets and health fairs and advertisements using local media (N= 280) | Sedentary adults recruited via self-referral (N = 239) | Sedentary adults recruited through newspaper advertisements (N=249). | Older adults recruited from health organisations (N = 1971). | Adults recruited from telephone company (N = 2827) |
| **Behaviour**  **Targeted** | PA (light-vigorous) | PA (moderate –hard activity) | PA (Moderate intensity to a level that met or exceeded ACSM recommendation) | PA (light-vigorous) | PA (Moderate) | PA (*Inferred -*light-vigorous)  Nutrition  Non Smoking |
| **Comparison group (s)** | Generic print (PA) controlled for contact time and number of materials. | Targeted info (PA: *choose to move)*;  General info (sleep, cancer prevention, nutrition).  *One mailing | Tailored telephone;  Generic print (attention control). | Tailored internet;  Standard internet | No info control  Environmentally tailored print | Generic print controlled for formatting and theoretical constructs |
| **PA primary outcome measures** | Self-report - adapted from seven-day PA recall questionnaire . | Seven-day PA recall questionnaire. | Seven-day PA recall questionnaire.  (sub sample used Actigraph to confirm validity) | Seven-day PA  *Secondary* fitness  actigraph (reported in methods but not in results paper) | PA: Self-report SQUASH questionnaire | PA: Self-report SQUASH questionnaire |
| **PA primary outcome variables** | Mins/wk  Meeting CDC/ACSM criteria | Mins/wk | Mins/wk  Meeting CDC/ACSM criteria | Mins/Week | Total weekly days of moderate PA  Meeting the recommendation for PA  In mediation paper (weekly minutes of PA – not taking intensity into account). | Mins/wk  Meeting the recommendation for PA |
| **Follow-up (post-baseline)** | One, three and 6 months  12 months (6 months post intervention). | 3 months  12 months  (6 months post intervention) | 6 months  12 months  (no post intervention measures) | 6 months  12 months  (no post intervention measures) | 3 months (during intervention; used to tailor final letter)  6 months  (approx 3 months post intervention, respectively) | 3 months  6 months  (during the intervention)  9 months  (3 months post intervention) |
| **Results** | Significant intervention effect for total PA at short-term and mid-term (145 mins/wk ±146.2 vs 102 mins/wk ± 98.3)..Trend at long-term (187 mins/wk. ± 216.1 vs 133 mins/wk ± 216.8) but no longer significant  Interventions effects for meeting the guidelines non significant at short term but significant at mid-term (45% vs 18%) and long-term (42% vs 25%). | Significant intervention effect at midterm compared to general info (140.35 mins/wk SE 14.82 vs 98mins/wk SE 15.09). Marginally significant intervention effect at midterm compared to targeted info (140mins/wk vs 100 mins/wk, SD not reported, p=0.054)  No significant differences at long-term but improvements across groups (tailored = 148.87 mins/wk, SE =19.13, targeted =154.48, Se =19.51, generic =139.52, SE = 19.61). | Significant intervention effects midterm for total PA (no difference between interventions: telephone = 123.32 mins/wk, SD =97.64; print = 129.49mins/wk, SD =156.46; contact-control =77.67 mins/wk, SD =101.79).  Significant intervention effects long -term for total PA (favouring print arm (162.37mins/wk, SD=165.17) – adverse effect for telephone arm (100mins/wk, SD =119.68).  Similar trend for meeting guideline.  Weak correlation at month 12 between PAR and actigraph (.32). | No significant intervention effects at mid (print: 112.5 mins/wk; t-internet=120 mins/wk; s-internet =90mins/wk) or long term (print: 90 mins/wk; t-internet=90 mins/wk; s-internet =80mins/wk). Increased PA in all groups and no significant differences between groups. | Significant intervention effects at midterm on both primary outcome variables (ES weekly days: S-tailored = 0.30; E-tailored: 0.35; OR guideline: S-tailored = 2.4; E-tailored: 2.8);  Difference between intervention arms differed via analyses [4, 5]  ES( mins/wk) small (0.35 at 6 months).  **Effect size reported** | Significant intervention effects at long-term for meeting the recommendation (*d* = 0.14) and total PA (*d* = .15).  No difference between tailoring conditions (actions plans no additional effect).  **Effect size reported.** |
| **Methodological Quality** | **Moderate**  Recruited via self referral  No intention to treat analysis with “moderately” scored withdrawal | **Moderate**  Recruited via self referral | **Moderate**  Recruited via self referral | **Moderate**  Recruited via self-referral  Actigraph data not reported  *Sample size based on power calculation (assuming a 30 minute difference between arms). | **Moderate**  Recruited via self-referral | **Weak**  High drop out. Differences between groups not described. |
| **Theory** | TTM  SCT  Decision theory | TTM  SCT  Decision  theory | SCT  TTM | TTM  SCT | I change;  TTM;  Health action process approach;  Self-regulation theory;  Self-determination theory | I Change |
| **Variables used for tailoring**  Key  Items were described *  Psychometrics given **  No description - | SOC **  Processes of Change **  Self efficacy**  Decisional balance**  PA score** | SOC**  Process of change **  Self efficacy **  Decisional balance **  Social support-  Goal setting-  PA score** | SOC**  Self-efficacy**  Decisional balance **  Processes of change**  PA score**  (mins/wk) | Refers to Marcus et al 1998 , 2007a [6, 7] | SOC-  Awareness *(calculated)*  Knowledge-  Attitude-  Self-efficacy-  Social influence-  Intention-  Intrinsic motivation-  Self regulation-  Action Planning-  Coping Planning-  PA score**  *plus condition:*  Perceived social environment (having a sports partner)-  Perceived physical environment – | Attitude **  Self-efficacy **  SOC **  Intention to implement action plans *  Barriers -  Social influence **  PA score**  (mins/wk) |
| **Operationalised?** | Yes  Examples of tailored messages given | Refers to Marcus et al 1998 [40] | Refers to Marcus et al 1998 [40] | Refers to Marcus et al 1998 , 2007a [40-41] | Yes, describes content per newsletter intext.  Determinants, theoretical methods, practical strategies provided as appendix | Yes  Describes what content was based on (e.g. message differed based on self-efficacy and attitude) but not theoretical strategy. |
| **Delivery method & delay** | Mail within 1 week after baseline | Mail - time not described | Mail- time not described | Mailed within 1 week | Mailed within 2 weeks | Mail -time not described |
| **Format of materials** | Tailored reports and stage based manuals | Tailored reports and stage based manuals | Tailored print + generic information package  + stage-based manual and monthly tip sheet | Tailored reports and stage based manuals  + general info about PA and tips for becoming active (to control for info available in internet arm) | Between 3-11 pages depending on changes in activity and determinants scores.  *Plus condition:*  + maps, cycle routes, phone numbers of sports clubs, ebudy weblink  Example layout given [5, 8]. | Tailored letters 5-11 pages  Cartoons, text and graphics |
| **Content of Print Materials** | Text with key messages highlighted  Descriptive feedback: psycho-social variables  Comparative feedback: Scores compared to successful individuals  Evaluative/Progress feedback: progress made on psycho-social and PA behaviour change since prior assessment | As in Marcus et al 1998 [40]  Descriptive  Comparative  Progress | Generic information package: how to take your heart rate, buying walking shoes, proper walking posture.  Tailored feedback based on psycho-social and behavioural variables  Comparative Feedback: Scores compared to successful individuals  Evaluative/Progress feedback: progress made on psycho-social and PA behaviour change since prior assessment | Tailored and targeted materials  Tailored feedback based on psycho-social and behavioural variables  Descriptive: feedback based on assessment of constructs  Comparative Feedback: Scores compared to successful individuals  Evaluative/Progress feedback: progress made on psycho-social and PA behaviour change since prior assessment | Evaluative: behaviour change strategies based on psycho-social and behavioural variables (and environmental in the *plus* condition).  Progress feedback (third letter): progress made on PA and determinants since baseline. Improvements rewarded and possible relapses addressed with additional advice. | Evaluative: PA score compared to self-assessed PA level and recommended minimum level. Misconceptions addressed. Behaviour change strategies based on psycho-social variables.  Progress: progress made on PA since last assessment.  Generic advice: social support  *For subset:* Action plans. |
| **Delivery Schedule** | baseline, 1m, 3m & 6m | baseline, 1m, 3m & 6m | Weekly for the first month. Biweekly for months 2 & 3; monthly for months 4-6. No contact in month 7, 9 and 11. Bimonthly in months 8,10,12. | Monthly for 12 months (*inferred)* | 2 weeks, 8 weeks, 14 weeks | Baseline, 3months, 6months |
| **Tailoring assessment period** | Just prior to Baseline, 1m, 3m,6m | Just prior to Baseline, 1m, 3m,6m | Monthly | Monthly | Baseline (Tailored letters 1 and 2)  3 months (letter three – addressing progress) | Baseline, 3months, 6months |
| **Interpretation of results based on theory?** | Mediation analysis  -No sig group differences observed for any psycho-social constructs even though significant power  Moderators – intervention more effective for those in the precontemplation and contemplation stage | No | SOC, Self-efficay – mediator  Process measures discussed | Based on SCT, TTM and empirical research picked variables that should impact study outcomes. Included conceptual model [9].  Moderators  Mediators | Intervention mapping protocol used to develop intervention.  Moderators [4] different primary outcome variable used. | Goal setting and implementation intention theories |
